# Genito-Urinary Surgery

**Published:** 1905-08-05

**Authors:** 


### GENITO-URINARY SURGERY.

**Decapsulation of the Kidneys.**—Francis D. Boyd[1] refers to the conclusions of Edebohls that decapsulation of the kidneys in chronic Bright's disease leads to the formation of new vascular connections between these organs and the surrounding parts and to improvement and restoration to health of the kidney. In the 72 cases operated upon by Edebohls seven deaths were immediately subsequent to the operation. Twenty-two patients died from causes not directly attributable to the operation and of these 13 died from chronic nephritis. Twenty patients experienced decided improvement. To treat chronic nephritis by improving the renal circulation seems eminently rational. The question at issue is: Does decapsulation result in such an improvement in the renal circulation? On this point observers differ. Johnson, experimenting on dogs, found that a new capsule was formed but without any considerable anastomosis between renal and peri-renal vessels. Hall and Herxheimer, having decapsulated the kidneys of animals in which acute nephritis had been set up, similarly obtained no evidence that the decapsulation had improved the renal circulation. Ferrarini found that decapsulation did improve the circulation. There are very few post-mortem records of the human subject which reveal the effect of the operation on the circulation in the kidney. Edebohls claims to have post-mortem evidence of the correctness of his views. Jewill reports a case in which the post-mortem showed no newly-formed blood-vessels passing into the kidney. Boyd here reports another post-mortem. He writes that the new capsule, though dense, was vascular, but "it is doubtful whether the formation of these new vessels could more than compensate for the normal communication between the renal and peri-renal vessels, which was ruptured by the operation of decapsulation." Decapsulation has been followed by diuresis and diminution of albumin in the urine and a rise in the urea excretion. This may be due to the relief of tension, but against this view is the fact that there has been no evidence at the operations, in many cases, of increased tension in the kidney. Possibly the operation stimulates the sympathetic centres in relation with the kidneys. The author concludes that incision of the kidney may be useful in cases where there is kidney tension and marked diminution in the amount of urine excreted, but neither decapsulation nor incision can be expected to cure chronic nephritis.

**Movable Kidney.**—David Newman[2] discusses the prognosis and treatment of this affection. The writer has not had a case where death has resulted from the displacement, Keppler claims two such. The disturbance and discomfort induced by movable kidney depends upon circumstances. Those who can take care of themselves suffer little, though the mental derangement commonly associated with the condition may unfit the patient for social life. The writer has seen several cases where insanity has resulted. Active life may be prevented by the pain of movable kidney. The majority of patients lose flesh and become anæmic and flabby. Often, too, enteroptosis, with chronic gastritis and dilatation of the stomach, become associated with movable kidney. The displacement may obstruct the outflow of urine from the kidney, or it may obstruct the renal circulation leading to degenerative or inflammatory changes in the pelvis or parenchyma of the organ. In the author's experience the "nervous" or "hysterical" cases often in the long run do well after nephrorrhaphy, though the immediate results of the operation may be disappointing. With reference to treatment the cases may be divided into five classes—namely:—(1) Those in which the displacement gives little or no discomfort; (2) those with pronounced gastro-intestinal symptoms and later with neurasthenia; (3) those in which ill-defined pain, generally abdominal, is the most marked symptom; (4) those in which the pain is distinctly renal and is associated with albuminuria, hæmaturia, hydronephrosis or pyonephrosis; and (5) those in which both local and reflex symptoms are marked. For the first class of cases the author recommends specially made corsets. In all classes rest is very essential, and in some cases it may effect a cure. The author condemns electricity, friction, cold baths, and massage as absolutely useless. For the fixation of the kidney the author states that it is necessary to produce granulation tissue round the organ, and for this purpose the fibrous capsule must be divided and its margins sutured to the wound and either a gauze plug or large drainage-tube must lie between the kidney and the deeper parts of the wound for ten or twelve days. Gilbert Barling,[3] in examining for movable kidney, places the palm of the hand in the loin and the thumb in front. If this fails to reveal the organ a bi-manual examination is made in the semi-prone and in the knee-elbow positions, and finally with the patient standing up and leaning slightly forwards on to the hand of the same side. Some authors have considered that if the kidney returns to the upper loin during expiration an operation should not be advised. Barling does not hold this view, and is guided by the degree of pain or inconvenience induced by the mobility of the kidney in deciding for or against operation. Hydrophrosis may be the cause of or be caused by a movable kidney. Gall-stone colic or the pain of relapsing appendicitis when the appendix runs high up behind the colon, may have to be distinguished from the pain due to mobility of the kidney. Occasionally intense migrainous headache is due to renal mobility. The author reports two such cases in which a cure was effected by operation. In other cases there is extreme mental depression and neurasthenia. It is suggested that repeated pulling on the renal plexus of the sympathetic may account for these affections. The author's experience with abdominal belts has not been satisfactory. For neurasthenic patients he recommends operation. Goelet's method of operating is liked by the author. In this method temporary silk-worm-gut sutures are employed, which penetrate the capsule of the kidney and the parietes. This operation fixes the kidney in a higher position than most of the other methods. The upper half of the kidney is decapsulated to secure better fixation.

[1] Edin. Med. Journ., April 1905, p. 337. [2] Glasg. Med. Jour., April 1905, p. 253.

*(To be continued.)*

